# Exercise in Adolescence Enhances Callosal White Matter Refinement in the Female Brain in a Rat Model of Fetal Alcohol Spectrum Disorders

**DOI:** 10.3390/cells12070975

**Published:** 2023-03-23

**Authors:** Katrina A. Milbocker, Ian F. Smith, Eric K. Brengel, Gillian L. LeBlanc, Tania L. Roth, Anna Y. Klintsova

**Affiliations:** Department of Psychological and Brain Sciences, University of Delaware, Newark, DE 19716, USA

**Keywords:** fetal alcohol, FASD, myelin, oligodendrocyte, exercise, corpus callosum, unbiased stereology, microscopy, gene expression, intervention

## Abstract

A total of 1 in 20 infants born annually are exposed to alcohol prenatally, which disrupts neurodevelopment and results in several disorders categorized under the umbrella term Fetal Alcohol Spectrum Disorders (FASD). Children and adolescents affected by FASD exhibit delayed maturation of cerebral white matter, which contributes to deficits in executive function, visuospatial processing, sensory integration, and interhemispheric communication. Research using animal models of FASD have uncovered that oligoglia proliferation, differentiation, and survival are vulnerable to alcohol teratogenesis in the male brain due in part to the activation of the neuroimmune system during gestation and infancy. A comprehensive investigation of prenatal alcohol exposure on white matter development in the female brain is limited. This study demonstrated that the number of mature oligodendrocytes and the production of myelin basic protein were reduced first in the female corpus callosum following alcohol exposure in a rat model of FASD. Analysis of myelin-related genes confirmed that myelination occurs earlier in the female corpus callosum compared to their counterparts, irrespective of postnatal treatment. Moreover, dysregulated oligodendrocyte number and myelin basic protein production was observed in the male and female FASD brain in adolescence. Targeted interventions that support white matter development in FASD-affected youth are nonexistent. The capacity for an adolescent exercise intervention to upregulate corpus callosum myelination was evaluated: we discovered that volunteer exercise increases the number of mature oligodendrocytes in alcohol-exposed female rats. This study provides critical evidence that oligoglia differentiation is difficult but not impossible to induce in the female FASD brain in adolescence following a behavioral intervention.

## 1. Introduction

The U.S. Centers for Disease Control and Prevention estimates that 1 in 20 infants is affected by prenatal alcohol exposure (AE) annually, creating one of the largest pediatric public health crises in America [1,2,3]. Prenatal AE may result in facial dysmorphologies (e.g., smooth philtrum, thin upper lip, and orbital hypertelorism) and cognitive and behavioral impairments including hyperactivity, reduced executive function, deficits with perceptual learning and abstract conceptualization, reduced visuospatial processing, disrupted language and mathematical learning development, impulsivity, and disrupted social behaviors [3]. The severity of an individual’s physical, behavioral, and cognitive deficits is primarily determined by the timing and amount of prenatal AE [4]. Thus, there exist several distinct disorders classified under the Fetal Alcohol Spectrum Disorders (FASD) continuum to diagnose children with known or suspected prenatal AE.

As a teratogen, alcohol has varying effects on neurodevelopment resulting from differences in the vulnerability of brain structures, neural tissues, and cell types during different phases of gestation. The detrimental impact of prenatal AE on neuronal proliferation, differentiation, survival, and function is well described, and it has been studied using animal models of FASD [5,6,7]. More recently, prenatal AE has been shown to impact glia proliferation and function. For example, prenatal AE has been shown to trigger the hyperactivation of the neuroimmune response to variable environmental stimuli throughout life via the hypersensitization of cytokine-producing microglia [8]. Moreover, alcohol teratogenesis induces apoptosis of oligodendrocytes (OL), the myelinating glial cells of the central nervous system. In addition to myelinating axons, OLs serve as neuroimmune targets and regulators via expression of immune receptors and the production of chemokines and cytokines, respectively [9,10]. AE-induced neuroimmune activation during fetal development disrupts the myelination of early cortical-hippocampal circuits in murine models of FASD [11]. Such alterations to the white matter milieu during development affect the onset and progression of processes involved in circuit refinement in adolescence, such as synaptic pruning and myelination [12].

Indeed, prenatal AE leads to hypomyelination of large white matter tracts in the adolescent and adult brain of affected individuals [13,14,15,16,17,18,19]. Corpus callosum (CC) axons begin crossing the midline by the 12th post-conceptual week [20,21] and callosal myelination begins during the third trimester of fetal development, with the majority of axonal myelination completed within the first two years of life [22]. However, the patterning of myelination is continually altered through adolescence to accommodate connectome refinement and thus remains sensitive to environmental factors [23,24]. The phases of neonatal and adolescent myelination are conserved across mammalian species. For example, the initial wave of myelination in the rat brain occurs between postnatal days (PD) 8–24, followed by the secondary wave of myelin refinement on PD 37–45 [25]. Incomplete growth and myelination of the CC is characteristic of late-term AE, and these impairments result in poor interhemispheric communication due to variations in the volume, length, thickness, and orientation of callosal fibers in childhood and adolescence [13,19,25,26,27,28,29,30,31]. Deficits in bimanual coordination (interhemispheric transfer), perceptual and verbal learning, IQ, and executive function have been associated with corpus callosum dysmorphology in individuals with FASD [28,32,33,34,35].

Reduced oligoglia lineage cell proliferation and maturation during the late postnatal period and early life have been identified as key mechanisms underlying observed delays in CC myelination across the lifespan [13,36]. AE during the brain growth spurt (BGS), a period of rapid synaptogenesis overlapping with the onset of myelination, occurs during the third trimester of human gestation or the first two postnatal weeks in rodents and is a particularly gliotoxic period [25,37]. Several studies using animal models of FASD have demonstrated that OL differentiation and survival are significantly reduced in the infant and adolescent brain following AE during the BGS [38,39,40]. In addition to changes in oligoglia cell proliferation and survival, the production of myelin basic protein (MBP) is decreased both immediately following exposure to ethanol [41] and long term [40] in preclinical models of FASD. Alterations to the patterning of myelin ensheathment around axons is one of the lasting neuroanatomical alterations resulting from alcohol teratogenesis that likely alters synaptic transmission, leading to maladaptive behaviors [42,43].

Specific mechanisms by which alcohol teratogenicity alters oligoglia proliferation, maturation, and protein production are yet to be discovered. However, many patterns of epigenetic modifications such as chromatin remodeling, DNA methylation, and activation of histone-modifying enzymes have been closely associated with oligoglia fate specification and differentiation [44], with class I histone deacetylases (HDACs) being highly involved in regulating the maturation of oligodendrocyte precursor cells (OPCs) in normative development [45,46]. Unique epigenetic signatures are the result of an organism’s experience with its environment and may be altered across the lifespan by positive and negative effectors. While the effects of prenatal AE on epigenetic modifiers have been studied previously [47], the effect of prenatal AE on the epigenetic modification of class I HDACs, to our knowledge, has not been investigated.

An environmental factor that can potentially be harnessed to restore myelination in adolescence is aerobic exercise. Recent neuroimaging studies have linked aerobic exercise with increased CC myelination and improved executive function in children and adolescents [48,49]. Although examination of the cellular mechanisms underlying these changes remains understudied, increased proliferation and maturation of oligoglia in the brain has been demonstrated in juvenile and adolescent rodents exposed to voluntary exercise regimes in adolescence and adulthood [50,51]. Moreover, increased voluntary aerobic activity has been shown to mitigate some of the alterations to neurogenesis in animal models of FASD [52]. This, along with the well-established associations of aerobic exercise with neurogenesis, synaptogenesis, neurotrophic factor release, and amelioration of AE-induced neuroimmune dysfunction makes aerobic exercise an important behavioral intervention to investigate [53,54].

This study addresses several persistent gaps in knowledge surrounding oligoglia proliferation and differentiation from infancy to adolescence following late-term AE using a rat model of FASD. To investigate this, a rat model of binge-like AE during the BGS was employed in this study and measures of oligoglia lineage cell proliferation and maturation, MBP production, and epigenetic regulation of HDAC and myelin-related genes were obtained across different stages of development from infancy to adolescence. In addition, a thorough investigation of how AE affects the trajectory of myelination in the female brain is lacking. Thus, we provide seminal evidence of the impact of sex as a biological factor in CNS myelination in the infant, juvenile, and adolescent brain using a preclinical model of FASD. In addition to these goals, this study explores the capacity for an adolescent exercise intervention to stimulate myelination in the adolescent FASD brain.

## 2. Materials and Methods

### 2.1. Rat Model of FASD

**Figure 1:**
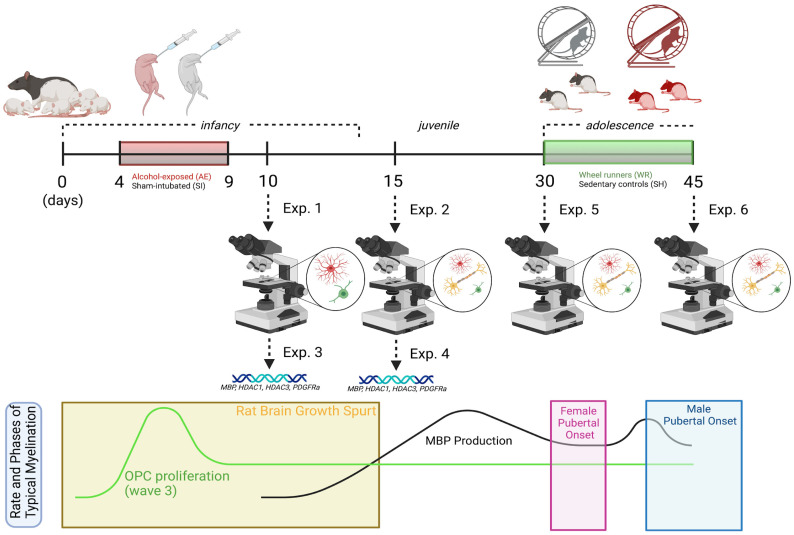
Experimental timeline with overlay illustrating the rate and phases of typical myelination in the rat brain from infancy to adolescence.

All rat acquisition, care, and methodology was in accordance with the guidelines provided by the University of Delaware’s Institutional Animal Care and Use Committee, the NIH Guide for the Care and Use of Laboratory Animals, and the ARRIVE guidelines (https://arriveguidelines.org, accessed on 1 January 2020). Timed-pregnant dams (Charles River Laboratories, Boston, MA, USA) were acquired and once pups were born, litters were culled to 10–12 pups each and counterbalanced for sex and postnatal treatment condition (AE or sham-intubated (SI) control). As shown in Figure 1, pups from 4–5 litters were obtained for Experiments 1, 2, and 5, and pups from 7–8 litters were obtained for Experiments 3, 4, and 6. On postnatal day (PD) 3, each pup received a small subcutaneous injection of India Ink in the paw and an ear punch for ease of identification across the animal’s lifespan. From PD 4–9, half of each litter received a daily dose of alcohol in milk-substitute at 5.25 g/kg/day (11.9% *v*/*v*, divided into two administrations two hours apart) resulting in an average blood alcohol concentration (BAC) of 300–400 mg/dL. This technique models a binge-like exposure during the BGS, as this neurodevelopmental milestone occurs during the third trimester of pregnancy in humans and the first two postnatal weeks in rats [55]. SI procedural control rats received intragastric intubation without liquid administration to account for the stress of intubation without altering the nutrient intake of typically-developing rats. Notably, this is a well-established model in the field of preclinical FASD research and it is known that the level of BAC achieved is correlated with region-specific apoptosis of neurons and OLs [5,6]. Following the dosing period, male and female pups were left to mature with a dam before sacrifice on PD 10 or 15 or after weaning on PD 30 or 45 (see Figure 1).

### 2.2. Adolescent Exercise Intervention to Stimulate Myelinogenesis

For experiments 5 and 6 depicted in Figure 1, male and female rats from both postnatal treatment groups were weaned on PD 23 and pair-housed in same-sex groups. On PD 30, rats from each group were randomly selected to either remain in social housing as sedentary controls (SH) or be rehoused into a modified cage with an attached running wheel for 24/7 voluntary access to the exercise intervention (wheel running, WR). Rats were sacrificed between PD 42–45 and brain tissue was collected for immunohistochemical analysis of the oligoglia lineage cell number and MBP densitometry (Exp. 6; n = 7–12). It should be noted that this cohort of rats is the same cohort that underwent longitudinal noninvasive neuroimaging scanning (the reason for the range of sacrifice days) as described in Milbocker et al. (2022) [56], and there is no evidence to suggest that the scanning protocol had any measurable effect on the cellular features quantified in this study. The circumference of the wheels was used to convert the number of rotations to distance run (kilometers). The average distances run for each cohort of Experiment 6 are outlined in Milbocker et al., 2022 [56]. An additional cohort of rats was generated and sacrificed on PD 30 to quantify these measures in brain tissue pre-intervention (Exp. 5; n = 8–12).

### 2.3. Animal Euthanasia for Tissue Collection

To obtain brain tissue samples for immunohistochemical and RT-PCR analysis, two euthanasia methods were required. For immunohistochemistry, rats were anesthetized using an intraperitoneal injection of a ketamine-xylazine mixture and inhalation of isoflurane prior to transcardiac perfusion with 100 mL of heparinized phosphate buffered saline (PBS, pH = 7.3) and 100 mL of 4% paraformaldehyde in PBS at 4 °C on PD 10, 15, 30, or 45 (Exps. 1, 2, 5, and 6). Brains were stored in vials containing 4% paraformaldehyde in PBS and swapped 3 times during fixation using a 30% sucrose in 4% paraformaldehyde in PBS mixture. For RT-PCR gene expression analysis, rats were sacrificed via rapid decapitation on either PD 10 or 15 (Exps. 3 and 4). Brains were quickly extracted and flash frozen with freezing isopentane. Frozen brains were sectioned using a 1 mm blocking matrix (product no. RBM 3000-C, ASI Instruments, Warren, MI, USA). Sections were stored on microscope slides at −80 °C until processing for gene expression.

### 2.4. Tissue Preparation for Oligoglia Cell Quantification

To uncover the immediate and lasting effects of AE during the BGS on oligoglia lineage cell proliferation and maturation, OPC and mature OL quantification was conducted at four postnatal time points. In the infant/juvenile stages, tissue samples were collected immediately following the dosing paradigm at the onset of forebrain myelination on PD 10 (Exp. 1; n = 10) and during the peak in rat CC myelination on PD 15 (Exp. 2; n = 10). In adolescence, tissue samples were collected pre- and post-exercise intervention, targeting a second, albeit smaller peak in forebrain myelination (PD 30 and 45, respectively; Exps. 5 and 6; n = 7–12).

Every eighth coronal section of the infant/juvenile corpus callosum (Exps. 1 and 2) and every sixteenth coronal section of the adolescent corpus callosum (Exps. 5 and 6) was used for immunofluorescent staining against the PDGFRα (OPC) and CC1 (OL) antigens and counterstained against Hoechst 33,342 to accurately identify oligoglia cell bodies. Free-floating tissue sections were rinsed with 0.1 M Tris-Buffered Saline (TBS; pH = 7.40) three times (5 min. per rinse) and blocked in blocking solution [0.1% Triton X-100 (BP-151; ThermoFisher Scientific; Hampton, NH, USA), and 5% Normal Donkey Serum (S30; Merck Millipore; Burlington, MA, USA) in 0.1 M TBS] for 2 h at room temperature. After blocking, tissue was incubated in primary antibody solution [1:400 mouse anti-APC/CC1 (OP-80; EMD Millipore, Burlington, MA, USA) and 1:500 rabbit anti-PDGFRα (SAB4502142; Sigma Aldrich, St. Louis, MO, USA) in blocking solution] for approximately 12 h at 4 °C in non-perforated bubble trays. Control sections were placed in blocking solution with no primary antibodies.

On day 2, tissue was rinsed in 0.1 M TBS three times (10 min. per rinse) and incubated in secondary antibody solution [1:400 goat anti-rabbit biotinylated antibody (30,014; Vector) in blocking solution] for 2 h at room temperature to increase visualization of OPCs. Following amplification, tissue was rinsed in 0.1 M TBS three times (10 min. per rinse) and incubated in fluorescent antibody solution [1:250 donkey anti-mouse antibody with Alexa Fluor 594 (A21203; ThermoFisher Scientific, Waltham, MA, USA) and 1:750 donkey anti-goat antibody with Alexa Fluor 488 (705-545-003; Jackson ImmunoResearch, West Grove, PA, USA) in blocking solution] for 2 h at room temperature. Following fluorescent antibody incubation, tissue was rinsed in 0.1 M TBS three times (10 min. per rinse) followed by 0.1 M PBS for 5 min. Tissue was then stained with Hoechst 33,342 (62,249; ThermoFisher Scientific, Waltham, MA, USA) for 5 min and rinsed with 0.1 M PBS three times (5 min. per rinse). Lastly, tissue was slide-mounted in dH2O and air-dried for 1 h before coverslips were mounted with Gelvatol. Coverslipped slides were stored away from light at 4 °C and were allowed to set for at least 12 h before microscopic analysis.

### 2.5. RT-PCR Analysis of Brain Tissue for the Identification of Epigenetic Modifications to Genes Relevant to Developmental Myelination

Disrupted proliferation and maturation of oligoglia lineage cells and myelin production in infancy have lasting consequences for circuit refinement in adolescence. Following the identification of alcohol-induced alterations to oligoglia lineage cell number in infancy, we sought to identify a potential neurobiological mechanism underlying these alterations in cell populations. Two additional cohorts of rats were generated to extract tissue samples for RT-PCR analysis of epigenetic modifications to genes associated with developmental myelination on PD 10 (Exp. 3; n = 12) and PD 15 (Exp. 4; n = 12). 

The rostral body of CC was dissected on dry ice and homogenized. DNA and RNA were extracted using an Allprep DNA/RNA Micro Kit (Qiagen) and stored at −80 °C. Nucleic acid quality was confirmed using spectrophotometry (NanoDrop 2000, ThermoFisher Scientific, Waltham, MA, USA). Reverse transcription was performed by converting RNA to cDNA using a cDNA synthesis kit (Qiagen). cDNA was amplified via RT-PCR (Bio-Rad CFX96, Hercules, CA, USA) using Taqman probes (Applied Biosystems) targeting *Mbp* (Assay ID: Rn01399617_m1), *Pdgfrα* (Assay ID: Rn01399472_m1), *Hdac1* (Assay ID: Rn01519308_g1), and *Hdac3* (Assay ID: Rn00670567_g1). Tubulin (Assay ID: Rn01435337_g1) was used as a reference gene, no differences of which were observed between experimental groups in these experiments. Reactions for all gene targets were run in triplicate and product specificity was verified using gel electrophoresis.

It should be noted that PDGFRα, a receptor for the potent OPC mitogen PDGF-AA, is widely regarded as the best characterized marker of pre-myelinating OPCs, although it may also be expressed in neurons and other glial cells in surrounding regions [57,58,59]. Thus, co-localization with an oligoglia-specific marker such as OLIG2 may avoid this potential limitation in future studies.

### 2.6. Tissue Preparation for Densitometric Analysis of Myelin Basic Protein

In addition to alterations in oligoglia cell population, MBP density was measured using densitometry on PD 15, PD 30, and PD 45 (Exps. 2, 5, and 6; n = 7–12). Every sixteenth coronal section containing the CC was immunohistochemically stained, conserving rostro-caudal order, with a primary antibody against MBP. The staining protocol was modified from Milbocker and Klintsova (2021) [60] and Hamilton et al. (2019) [61]. Free floating sections were rinsed in a series of 0.1 M TBS and quenched in 0.4% Triton X-100 with 0.6% H2O2 in TBS for 30 min. After additional rinses of TBS wash, the sections were placed in blocking solution [4% Normal Goat Serum (NGS) and 0.4% Triton X-100 in 1× TBS] for one hour. Following blocking, the sections were transferred into non-perforated bubble trays filled with primary antibody solution [Rat anti-MBP antibody (MAB386, Millipore) at 1:200 dilution in blocking solution]. Control sections were placed in the blocking solution with no antibody. 

Approximately 48 h later, the sections were transferred from the non-perforated bubble trays and placed in TBS wash. They were then incubated in secondary antibody solution for 2 h [Biotinylated goat anti-rat secondary antibody (BA9400, Vector) 1:1000 dilution in blocking solution]. After an additional TBS rinse, the tissue was placed in an avidin-biotin complex (ABC) amplification solution in blocking solution for 60 min. Another TBS rinse was followed by the placement of tissue sections in 0.05% 3,3′-Diaminobenzidine (DAB)-Peroxidase with nickel enhancement [2 mL DAB + 1.39 g nickel ammonium sulfate + 200 mL 1X TBS] for an average of 8.83 min. Once sufficiently stained, the tissue was placed in a final TBS rinse and mounted onto gelatin-subbed microscope slides. 48 h later, and the slides underwent a series of brief rehydration/dehydration washes (30–60 s each) before being cover slipped using DPX Mountant.

### 2.7. Stereological Estimation of Cell Number and Densitometry

For systematic, unbiased estimation of OPC and OL quantity and region volume through the entire body and splenium of the corpus callosum, the StereoInvestigator Optical Fractionator probe (MBF Bioscience, Williston, VT, USA) was used (6–11 sections per brain). Tissue images were acquired on a Zeiss AxioImager M2 microscope (Carl Zeiss AG, Oberkochen, Germany) equipped with a high-sensitivity monochrome camera (ORCA-Flash4.0 LT+ Digital CMOS Camera, Hamamatsu Corporation, Middlesex, NJ, USA) with the region of interest contoured at 5X magnification. Individual cells were then quantified at 40X objective magnification with stereological parameters sufficient to obtain average coefficients of error < 0.10 (grid size = 274 μm × 274 μm; counting frame = 150 μm × 150 μm; region of interest percent quantified = 30%) [62]. After quantification, estimates of total OPC and OL populations and CC volume were calculated within the Optical Fractionator workflow of the StereoInvestigator software.

Optical densitometry was measured as an analog for assessing the amount of MBP in the body and splenium of the CC, using a protocol modified from Gursky et al. (2020) [52] and Purohit et al. (2020) [63]. The CC was traced on the mounted tissue sections at 5X and imaged at 20X magnification using a Zeiss Axioskop2Plus microscope (Carl Zeiss AG, Oberkochen, Germany) and Stereo Investigator software (MBF Bioscience, Williston, VT, USA). Images were acquired in gray scale and white balance was performed. The number of CC tracings per animal ranged from 5–8. Mean optical density values were recorded for every section per animal using the optical density calibration protocol provided by ImageJ (version: Java 8, National Institutes of Health, Bethesda, MD, USA). Following calibration, all values ranged from 0 to 2.6, and mean optical density values of each section of the CC were thresholded by subtracting values from the CC from values in a region of the cortex to account for background staining. Densitometric analysis of MBP within the body and splenium of CC were assessed separately as the splenium has been shown to be extremely vulnerable to prenatal AE [43].

### 2.8. Statistical Analysis

Analysis of histological and gene expression data was performed using two-way ANOVA with postnatal treatment X adolescent condition as independent factors or nonparametric Kruskal Wallis tests when appropriate (IBM SPSS statistical software, version 27, Armonk, NY, USA). Levene’s Test for homogeneity of variances was run on each dataset a priori to determine the appropriate statistical test required to analyze each dataset. Outliers falling outside of 1.5 times the interquartile range were discarded (max 3/group), and post hoc analyses were performed with Bonferroni correction for multiple comparisons. For comparison of cellular parameters measured on PD 30 (pre-intervention), sex was included as an independent variable in the analysis. To compare similar parameters on PD 45 (post-intervention), the data for each sex were analyzed separately to account for sexual dimorphisms in the data (n = 8–11) [64]. Finally, to compare changes to the oligoglia cell number, MBP density, and gene expression between time points, independent or one-sample t-tests were employed within treatment groups.

## 3. Results

### 3.1. Estimation of Oligoglia Number in the Body of Corpus Callosum from Infancy to Adolescence

**Figure 2:**
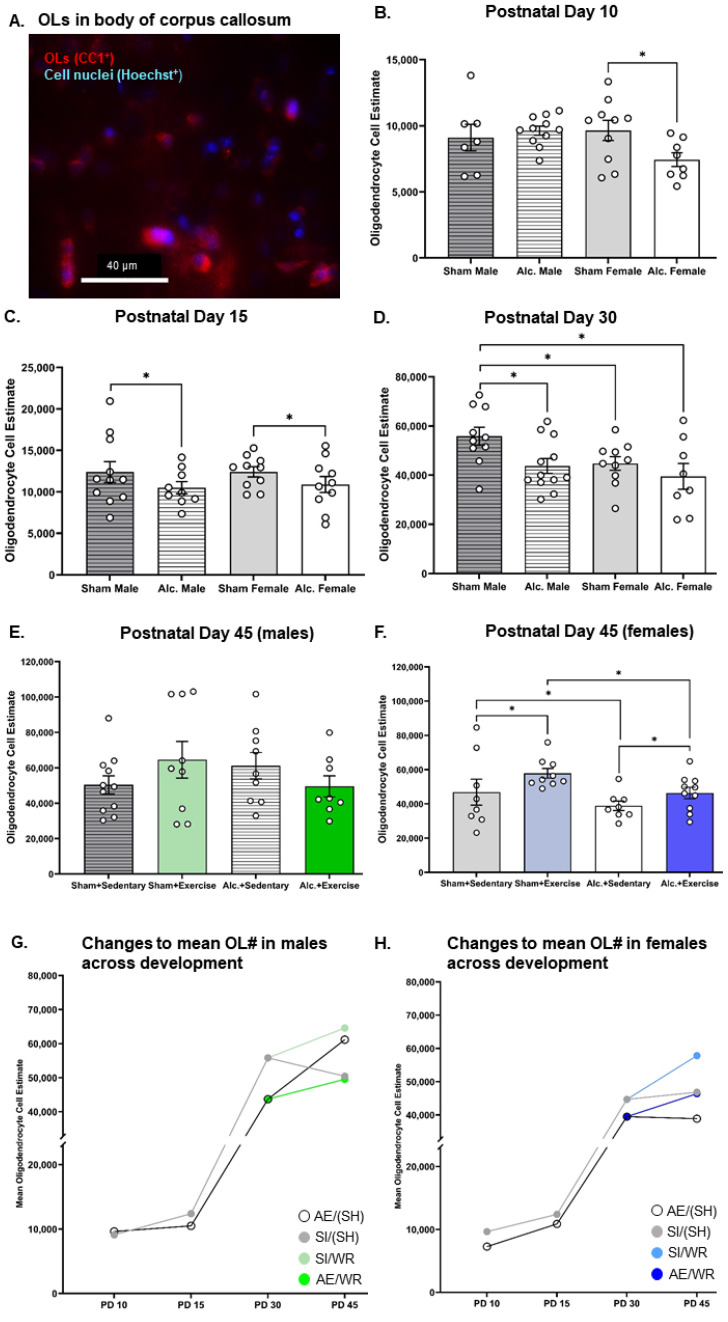
OL quantification in the body of CC from PD 10 to 45. (**A**) Example of fluorescent staining used to visualize OLs in CC, scale bar = 50 µm; (**B**–**D**) Comparison of the number of OLs in the body of CC in brain tissue extracted from AE and SI rats from both sexes; (**E**,**F**) Comparison of the number of OLs in the body of CC in SI/SH, SI/WR, AE/SH, and AE/WR male (**E**) and female (**F**) rats. Each data point in graphs B-F represents the mean estimate per rat and error bars represent ±SEM, * *p* ≤ 0.05; (**G**,**H**) Trajectories of OL differentiation across all time points in the male (**G**) and female (**H**) brain in all postnatal treatment and intervention groups.

There were significant main and interactive effects of the postnatal treatment group on the number of myelinating OLs in the body of CC from infancy to adolescence that differed between sex. Two-way ANOVA indicated that there was no main effect of the postnatal treatment condition (*p* = 0.93) or sex (*p* = 0.57) on OL population estimate, with a significant crossover interaction of postnatal treatment condition and sex (F_4,30_ = 5.7, *p* = 0.02, η^2^ = 0.16) on PD 10. Pairwise comparisons confirm that the OL number was more vulnerable to third trimester-equivalent AE in the female brain at this time (Figure 2B). On PD 15, postnatal treatment significantly altered the OL population estimate (Figure 2C). Analysis using a two-way ANOVA indicated that there was a main effect of postnatal treatment condition (F_4,35_ = 4.2, *p* = 0.047, η^2^ = 0.11) but not sex (F_4,35_ = 2.1, *p* = 0.15) on the OL population estimate, with no significant interactions. *Post hoc* analysis confirmed that the OL number was reduced in the AE male and female brain on PD 15.

Prior to intervention exposure, female rats from both postnatal treatment groups had the lowest number of OLs in CC compared to their male SI control counterparts (F_1,36_ = 4.5, *p* = 0.04, η^2^ = 0.11; Figure 2D). AE reduced the number of OLs in the male brain such that it was comparable to the number of myelinating glial cells in the SI female brain on PD 30 (F_1,36_ = 5.7, *p* = 0.02, η^2^ = 0.14; Figure 2D). AE female rats that did not receive exercise intervention exhibited a reduction in the number of OLs on PD 42 (F_1,31_ = 4.8, *p* = 0.04, η^2^ = 0.14, Figure 2F). However, when provided with voluntary access to running wheels for twelve consecutive days, the number of OLs rose in the female brain to control levels (F_1,31_ = 4.4, *p* = 0.045, η^2^ = 0.12). The highest number of OLs was found in the intervention-exposed SI female brain (Figure 2F). The number of OLs in the male brain did not differ between groups post-intervention (Figure 2E), indicating that alcohol-induced reductions to the OL population were transient. These findings are convergent with those described by Newville and colleagues (2017) [40].

**Figure 3:**
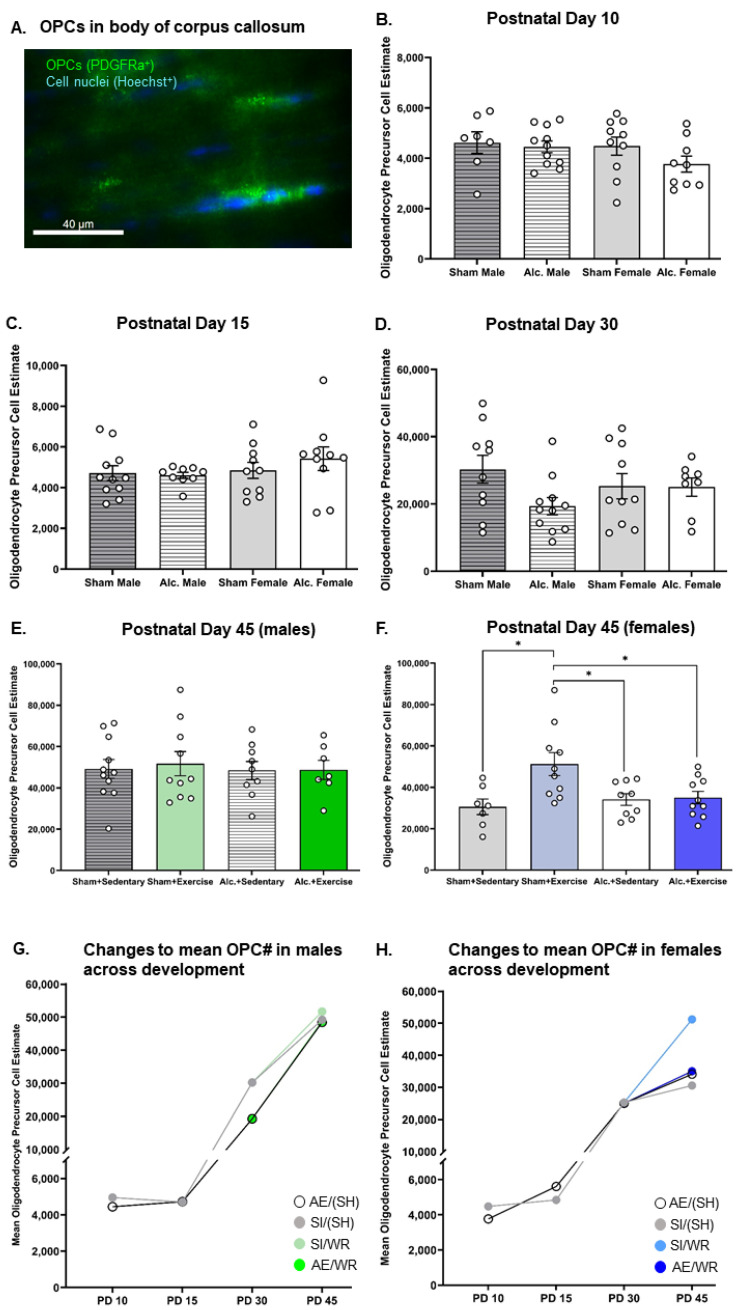
OPC quantification in the body of CC from PD 10 to 45. (**A**) Example of fluorescent staining used to visualize OPCs in corpus callosum, scale bar = 50 µm; (**B**–**D**) Comparison of the number of OPCs in the body of CC in brain tissue extracted from AE and SI rats from both sexes; (**E**,**F**) Comparison of the number of OPCs in the body of CC in SI/SH, SI/WR, AE/SH, and AE/WR male (**E**) and female (**F**) rats. Each data point in graphs B-F represents the mean estimate per rat and error bars represent ±SEM, * *p* ≤ 0.05; (**G**,**H**) Trajectories of OPC proliferation across all time points in the male (**G**) and female (**H**) brain in all postnatal treatment and intervention groups.

Conversely, neither postnatal treatment condition nor sex significantly altered OPC population estimates on PD 10 (*p* > 0.05; Figure 3B). Further, group differences in OPC number were not observed on PD 15 (*p* > 0.05; Figure 3C). Moreover, the estimated number of OPCs in the body of corpus callosum remained unaffected by AE at pre- and post-intervention time points, irrespective of sex (*p* > 0.05; Figure 3D–F). However, the number of OPCs was increased following intervention exposure in female SI control rats only (F_1,32_ = 5.8, *p* = 0.02, η^2^ = 0.15; Figure 3F). Since a similar effect of intervention exposure was not identified in AE female rats, these data suggest that AE during the BGS may limit the capacity for OPC proliferation in response to external stimuli in white matter regions of the adolescent female brain.

### 3.2. Densitometric Analysis of MBP Production in the Body and Splenium of Corpus Callosum from Late Infancy to Adolescence

**Figure 4:**
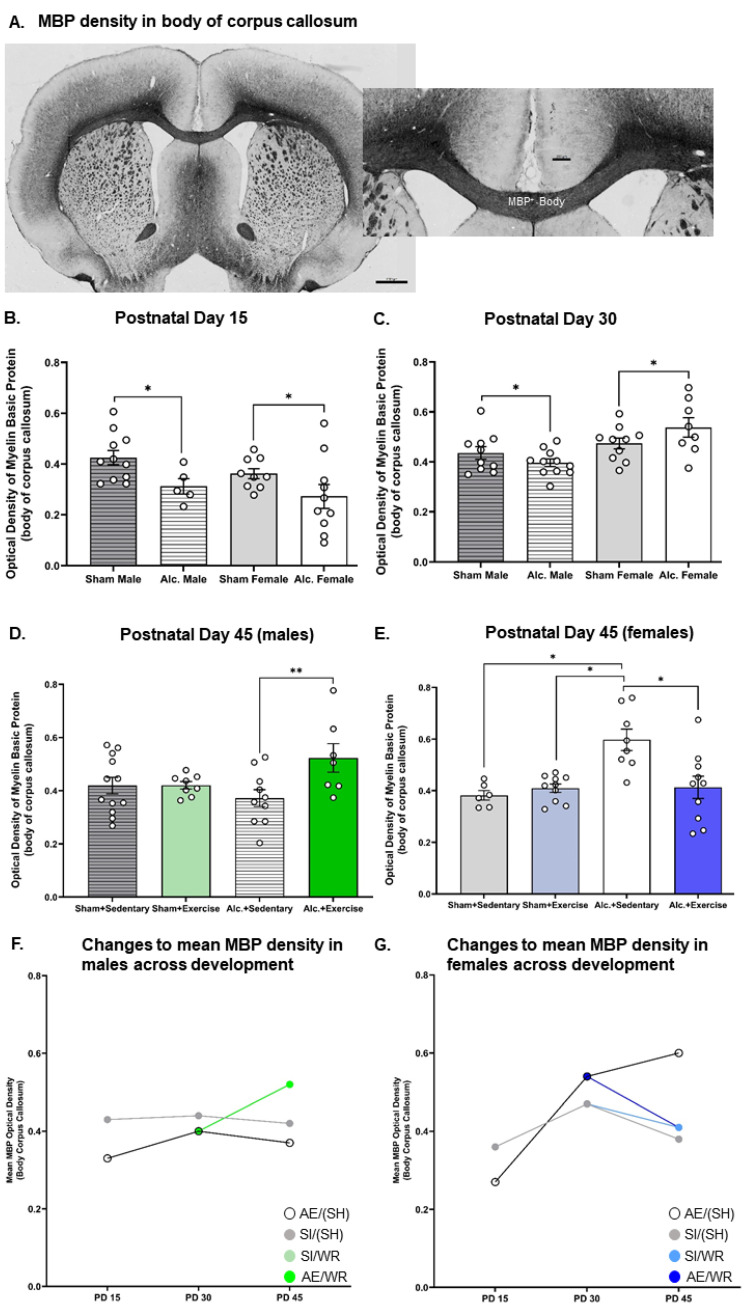
Densitometric analysis of MBP in the body of CC from PD 15 to 45. (**A**) Example of histological staining used to visualize MBP in corpus callosum, scale bar = 1–2 mm; (**B**,**C**) Comparison of the optical density of MBP in the body of CC in brain tissue extracted from AE and SI rats from both sexes; (**D**,**E**) Comparison of the optical density of MBP in the body of CC in SI/SH, SI/WR, AE/SH, and AE/WR male (**D**) and female (**E**) rats. Each data point in graphs (**B**–**E**) represents the mean estimate per rat and error bars represent ±SEM, * *p* ≤ 0.05, ** *p* ≤ 0.01; (**F**,**G**) Trajectories of MBP production across all time points in the male (**F**) and female (**G**) brain in all postnatal treatment and intervention groups.

**Figure 5:**
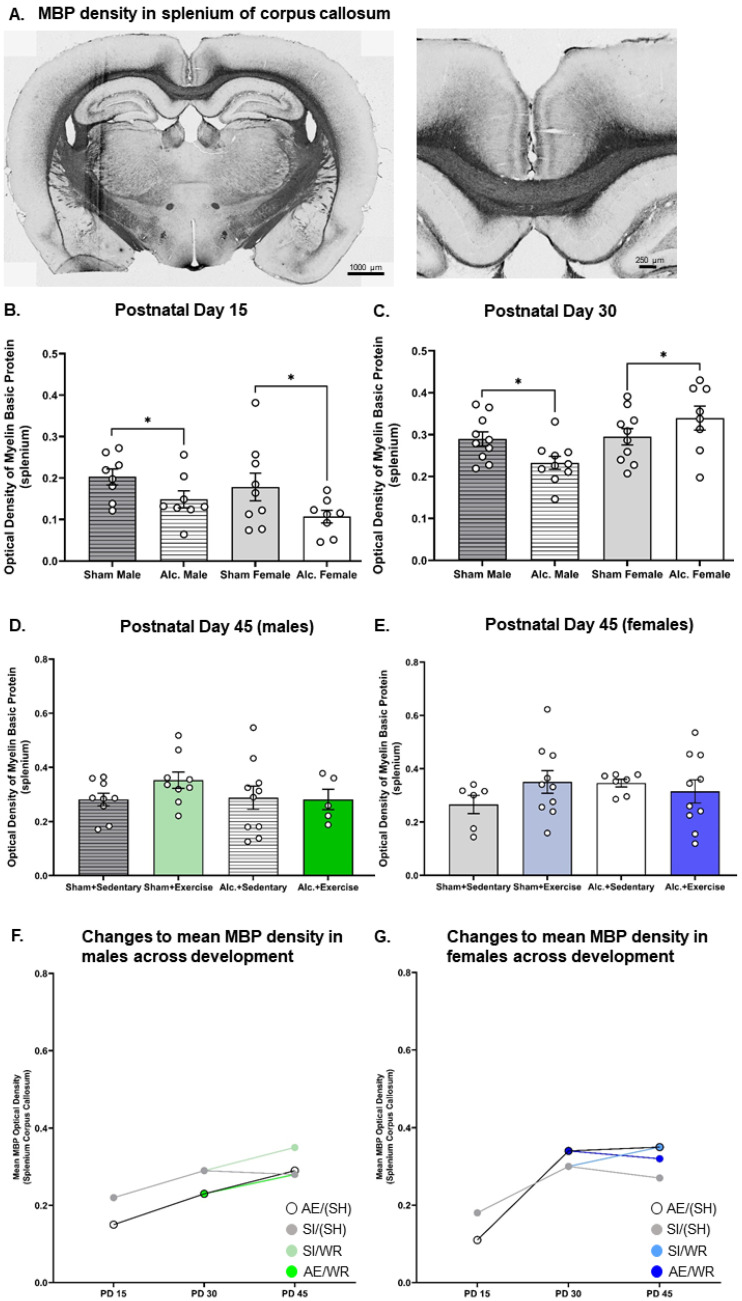
Densitometric analysis of MBP in the splenium of CC from PD 15 to 45. (**A**) Example of histological staining used to visualize MBP in corpus callosum, scale bar = 2 mm (left image) and = 0.75 mm (right image); (**B**,**C**) Comparison of the optical density of MBP in the splenium of CC in brain tissue extracted from AE and SI rats from both sexes; (**D**,**E**) Comparison of the optical density of MBP in the splenium of CC in SI/SH, SI/WR, AE/SH, and AE/WR male (**D**) and female (**E**) rats. Each data point in graphs B–E represents the mean estimate per rat and error bars represent ±SEM, * *p* ≤ 0.05; (**F**,**G**) Trajectories of MBP production across all time points in the male (**F**) and female (**G**) brain in all postnatal treatment and intervention groups.

MBP production in the body and splenium of the CC was reduced on PD 15 in the AE brain. Analysis with two-way ANOVA demonstrated a main effect of postnatal treatment on MBP optical density in the body (F_1,34_ = 7.86, *p* = 0.008, η^2^ = 0.19) and the splenium (F_1,29_ = 6.29, *p* = 0.013, η^2^ = 0.19) of CC. Post hoc analysis confirmed that AE during the BGS reduced MBP density in both sexes (Figure 4B and Figure 5B).

Similarly, densitometric analysis of MBP via comparison of optical density was investigated pre- and post-intervention. MBP concentration was demonstrated to be reduced in adolescent and adult male mice previously exposed to alcohol during the BGS [40,43]. The reduction in MBP concentration resulted in impaired axonal ensheathment by OL lamellae in the splenium subregion of CC. Thus, examination of MBP density in the whole body of CC and the splenium subregion was performed in our study. We found that on PD 30, there was a significant interaction between sex and the postnatal treatment group on the optical density of MBP in both subregions (Figure 4C and Figure 5C). AE increased MBP density in the female brain while the same exposure reduced MBP density in the male brain (F1_,35_ = 4.3, *p* = 0.046, η^2^ = 0.11). MBP production is upregulated following pubertal onset, and these data suggest that AE during the BGS may lead to precocial pubertal onset in females. Following exercise intervention, there was a significant interaction between postnatal treatment and intervention exposures on MBP density in the female body of CC. MBP density was highest in AE/SH rats and was restored to control levels by exercise intervention exposure (F_1,30_ = 9.23, *p* = 0.005, η^2^ = 0.24; Figure 4E), suggesting that the maturation of CC is stimulated by targeted adolescent intervention. Acute benefits of adolescent intervention on myelination of the whole body of CC are observed in female rats.

Moreover, there existed a significant treatment by intervention interaction on MBP production in the body of the CC in male rats post-intervention (F_1,36_ = 4.1, *p* = 0.05, η^2^ = 0.10; Figure 4D). Post hoc analysis revealed that MBP density was increased in AE rats that received intervention exposure and was not different between rats from either postnatal treatment condition that did not receive intervention exposure (*p* = 0.01). Contrary to our original hypothesis, we did not observe any main or interactive effects of postnatal treatment or intervention exposure on MBP density in the splenium subregions of CC in either sex on PD 45 (*p* > 0.05; Figure 5D,E).

### 3.3. Volumetric Changes to Body of Corpus Callosum from Infancy to Adolescence

**Figure 6:**
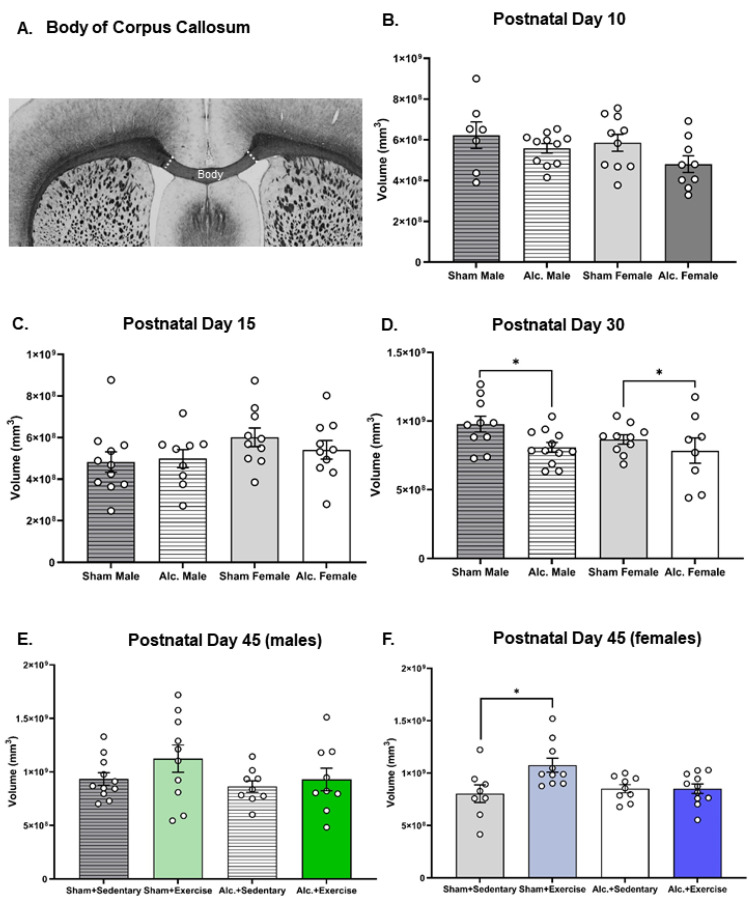
Volumetric analysis of the body of CC in all treatment and intervention groups from PD 10 to 45: (**A**) Macroscopic image of the body of CC, scale bar = 1 mm; (**B**–**D**) graphs illustrating the estimates of the volume of the body of CC for all groups on PD 10 (**B**), 15 (**C**), and 30 (pre-intervention; (**D**)); (**E**,**F**) graphs illustrating the estimates of the volume of the body of CC for male (**E**) and female (**F**) rats on PD 45 (post-intervention). Each data point represents the mean estimate per rat and error bars represent ±SEM. * *p* ≤ 0.05.

There were no significant differences between groups in the volume of the body of CC identified on PD 10 or 15 (Figure 6B,C). Volumetric analysis of the body of CC pre- and post-intervention indicated that AE limited the growth of this structure in adolescence. We found that AE rats from both sexes exhibited a smaller body of CC pre-intervention on PD 30 (F_1,36_ = 5.4, *p* = 0.03, η^2^ = 0.13; Figure 6D). There is a significant interaction between postnatal treatment and intervention exposure on CC volume on PD 42 in the female brain (F_1,34_ = 5.2, *p* = 0.03, η^2^ = 0.13; Figure 6F). Post hoc analysis confirmed that CC volume increased in female SI rats exposed to the intervention, but not in their AE counterparts. No such differences were observed in the male brain post-intervention (Figure 6E). These results are partially divergent from volumetric analysis of this subregion using noninvasive neuroimaging [56], and such discrepancies may result from either a lack of resolution in the in vivo scans or from the robust post-fixation process that leads to tissue shrinkage [65].

### 3.4. Analysis of Alcohol and Sex-Related Modifications to the Epigenome of Myelin-Relevant Genes

**Figure 7:**
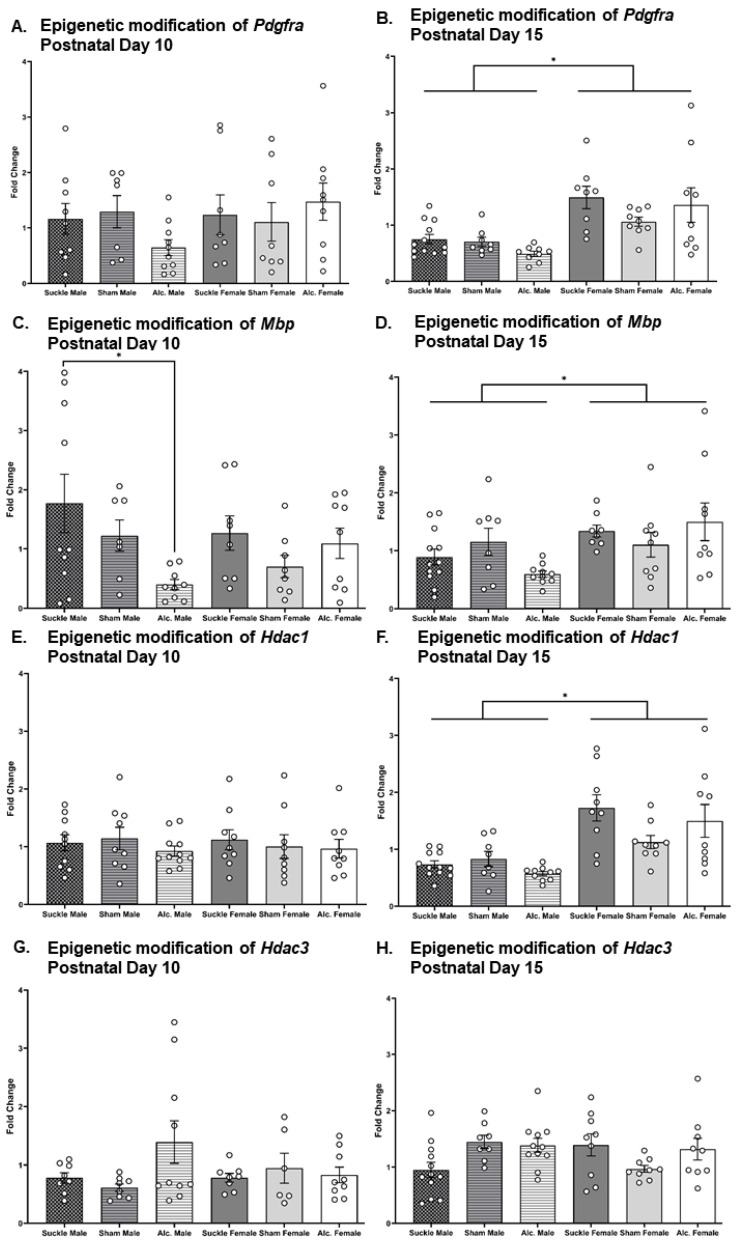
Fold change of *Pdgfra*, *Mbp*, *Hdac1*, and *Hdac3* genes on PD 10 relative to that observed in suckle control rats from analogous groups (**A**,**C**,**E**,**G**, respectively) and 15 (**B**,**D**,**F**,**H**, respectively). * *p* ≤ 0.05.

Two-way ANOVA were used to analyze the effects of postnatal treatment and sex on the expression of myelin-related genes *Mbp*, *Pdgfra, Hdac1*, and *Hdac3*. A significant main effect of postnatal treatment was found on *Mbp* expression in CC on PD 10 (F_2,49_ = 3.43, *p* = 0.043, η^2^ = 0.13). Post hoc analyses revealed a significantly lower expression of *Mbp* in male AE rats compared to male suckle control rats (*p* = 0.04; Figure 7C). There were no additional significant differences in gene expression observed on PD 10. On PD 15, there was a significant main effect of sex found on gene expression of *Mbp* (F_1,50_ = 7.49, *p* = 0.01, η^2^ = 0.13), *Pdgfra* (F_1,50_ = 26.89, *p* < 0.0001, η^2^ = 0.35), and *Hdac1* (F_1,51_ = 31.60, *p* < 0.0001, η^2^ = 0.38) in corpus callosum. An examination of group means revealed that female rats had greater expression of *Pdgfra*, *Mbp*, and *Hdac1* than male rats, regardless of postnatal treatment (Figure 7B,D,F).

We used *t*-tests to compare the trajectories of gene expression across development in the CC between groups (Table 1). The only significant difference in gene expression observed between PD 10 and PD 15 was a reduction to *Hdac1* expression in suckle control males. Overall, these analyses demonstrated that the expression of *Mbp*, *Pdgfra*, *Hdac1*, and *Hdac3* remains relatively consistent from PD 10 to PD 15, regardless of sex or postnatal treatment.

### 3.5. Comparing the Trajectories of OPC Proliferation, OL Differentiation, and MBP Production in AE and SI Rats from Each Sex

We used *t*-tests to compare trajectories of OPC proliferation, differentiation of OPCs to OLs, and of MBP production across development in the CC between groups (Table 2). As expected, trajectories of white matter maturation were sexually dimorphic and were, in some cases, sensitive to AE during the BGS and/or adolescent intervention. In male rats, from all postnatal treatment groups, OPC and OL populations remained static between PDs 10 and 15 (Figure 2G and Figure 3G). However, between PDs 15 and 30, CC maturation was expedited in the male SI control brain: there was a 542% increase in OPC proliferation and a 350% increase in the number of differentiated OLs (Figure 2G and Figure 3G). MBP production was upregulated by 32% in the splenium of male rats (Figure 5F). OPC proliferation continued to increase between PD 30 and 45 (71%) in the SI male brain and was unaffected by WR intervention, presumably to establish the vital OPC pool (Figure 3G). OL differentiation and MBP production were unchanged in the adolescent SI male brain (Figure 2G and Figure 4F). Conversely, AE severely limited the production of OPCs (308% increase), moderately reduced the differentiation of OLs (316%), and stimulated MBP production in both the body (21%) and splenium (53%) of CC from PD 15 to 30 in male rats (Figure 2G, Figure 3G, Figure 4F and Figure 5F). Acting as a potential compensatory mechanism, the number of OPCs produced was doubled in the AE male brain compared to that in the SI brain (151%) and OLs continued to differentiate (40%) in the AE/SH brain between PD 30 and 45 (Figure 2G and Figure 3G). Adolescent intervention exposure prevented OL differentiation but did not mitigate abnormal OPC proliferation in the male AE/WR brain from PD 30 to 45 (Figure 3G).

Similar analysis of the trajectory of white matter maturation in the female brain highlighted several fundamental differences in development of the body and splenium of CC compared to their male analogues in the SI and FASD brain (Table 2). One pivotal divergence included the elevated differentiation of OLs (29%) between PD 10 and 15 in the SI control female brain (Figure 2H). Similar to the male brain, OPC production (421%) and OL differentiation (260%) were significantly upregulated between PD 15 and 30 in these control rats (Figure 2H and Figure 3H). MBP production was also increased in the body (31%, Figure 4G) and splenium (67%, Figure 5G) of CC during this juvenile period in SI females. Neither OPC proliferation nor OL differentiation was significantly increased in the SI brain of rats without intervention exposure between PD 30 and 45 (Figure 2H and Figure 3H). Notably, OPC proliferation and OL differentiation was immediately stimulated by WR intervention in the female brain (103% and 29%, respectively; Figure 2H and Figure 3H). MBP density in the body of CC was reduced in female SI/SH (−19%) and SI/WR (−13%) rats during this period (Figure 4G). However, AE during the BGS impacted postnatal proliferation of OPCs (50%) and doubled the differentiation of OLs (50%) in CC compared to that in the SI control brain between PD 10 and 15 (Figure 2H and Figure 3H). Between PDs 15 and 30, OPC production was reduced (346%) and MBP production was significantly upregulated in the body (100%) and splenium (209%) of CC (Figure 3H, Figure 4G and Figure 5G). Importantly, restriction of MBP production observed in the body of CC in the SI female brain between PD 30 and 45 was not discovered in the AE brain (Figure 4G). Instead, OPC production was stimulated in female AE/SH rats during this time period (36%, Figure 3H). WR intervention mitigated this effect on MBP production in the body of CC in female AE/WR rats (−24%), but did not lead to a significant increase in OPC proliferation or OL differentiation like that observed in the female SI/WR brain (Figure 2H, Figure 3H and Figure 4G).

### 3.6. Correlation of Ex Vivo Analysis of Corpus Callosum Maturation with Previous In Vivo Neuroimaging Findings

As mentioned, the cohort of rats used in Experiment 6 was also scanned pre- and post-intervention using a diffusion tensor imaging protocol to noninvasively track changes to white matter maturation [56]. Radial diffusivity values extracted from diffusion tensor imaging scans describe the movement of protons perpendicular to white matter bundles. We previously showed that AE during the rat BGS elevated the radial diffusivity of protons moving perpendicular to CC fiber bundles when measured in the adolescent brain. Exercise intervention had no immediate effect on radial diffusivity in either SI or AE rats from both sexes. However, aerobic activity is a known effector of myelin cytoarchitecture [50,51]. Thus, it was predicted that microscopic alterations to CC myelination might be observed immediately following intervention, indicating a lack of sensitivity to structural changes in our in vivo scanning protocol.

Two-tailed partial correlation analysis controlling for treatment and intervention revealed that MBP density was not correlated with radial diffusivity in the female brain post-intervention. However, we did discover that these measures were positively correlated in the male brain post-intervention (R = 0.50, *p* = 0.02). This is likely due to the fact that exercise intervention mitigated the impact of alcohol teratogenesis on MBP density in the female adolescent brain, but a similar effect was not identified when comparing radial diffusivity values between groups. Moreover, several cellular features contributing to changes in myelination are changed in the female brain post-intervention. Conversely, the positive correlation between MBP density and radial diffusivity in the male brain suggests that changes to MBP density within-rat have corresponding changes to radial diffusivity. It should be noted that MBP density is the only myelin-related measure obtained that was changed following exercise intervention in the male body of CC. However, this effect was only demonstrated in the AE brain.

## 4. Discussion

The central goals of this study were to describe cytoarchitectural changes to CC development occurring from infancy to adolescence in the FASD brain and investigate a potential targeted exercise intervention to stimulate white matter maturation in the male and female FASD brain. The results confirm that this rat model of FASD delays white matter development in a similar manner to that observed in youth with late-term prenatal AE. Second, findings from this study contribute meaningfully to our understanding of the etiology of FASD in the female brain as it was among the first to examine the effect of prenatal AE on forebrain myelination in female rats. Third, this work identified several myelination-specific cellular targets that are immediately ameliorated by an adolescent exercise intervention. 

CC myelination begins during the BGS and extends into young adulthood for refinement of the adult connectome. The majority of axonal myelination in CC is complete within the first two years of life; however, myelin sheath development continues through adolescence, when a second myelination peak occurs, following synaptic pruning and connectome refinement. Developmental myelination ceases around 40 years of age, yet myelin ensheathment is continually altered to optimize function of specific neural circuits in adulthood [66]. The trajectory of white matter maturation is relatively conserved across mammalian species. Thus, a comprehensive investigation of the effects of AE during the BGS on white matter development from infancy to adolescence was facilitated with the use of a rat model of FASD in this study. We have recently conducted a neuroimaging study which suggested that the trajectory of CC myelination is delayed in AE rats irrespective of sex [56]. Specifically, radial diffusivity values, obtained with longitudinal Diffusion Tensor Imaging scanning, remained elevated in the CC of AE adolescent male and female rats, suggesting that axons were hypomyelinated. These findings were in unison with results from a study that demonstrated that AE during the BGS reduced CC myelination in adolescent male mice, as evidenced by elevated radial diffusivity values when measured in white matter in vivo [40].

As mentioned, environmental stimuli play a key role in optimizing circuit function via alterations to axonal myelination in adolescence. Aerobic exercise is a positive effector of central nervous system myelination in juvenile mammals as it stimulates the maturation of OLs [50,67,68]. Complex motor learning stimulates OPC proliferation in specific regions of the brain involved in motor control [69,70]. Further, one study conducted in mice demonstrated that 2 weeks of voluntary aerobic activity promotes myelination of axons in the motor cortex via Wnt signaling in adulthood. Specifically, voluntary wheel running led to an increase in the quantity of OPCs and myelinating OL, increased the production of MBP, and reduced the g-ratio (ratio between the diameter of an axon and the total diameter of a myelinated fiber), demonstrating that aerobic activity increased the thickness of the myelin ensheathment of axons in addition to stimulating oligoglia proliferation and differentiation. Notably, all mice used in this study were male [71].

As white matter maturation is one of the longest developmental processes in the mammalian brain, the use of an established rat model of FASD was integral to evaluate the effect of aerobic exercise on myelin plasticity in the adolescent FASD brain. Our lab and others have previously demonstrated that adolescent exercise intervention via free access to running wheels stimulates hippocampal and cortical neuroplasticity in a rat model of FASD [72,73,74,75]. However, research regarding the influence of aerobic activity on white matter tract myelination and OL morphology in rodent models of FASD is limited.

To validate our previous neuroimaging findings and to discover nuanced exercise-induced alterations to the cytoarchitecture within the OL matrix, the present study included two separate cohorts of rats to provide tissue samples for the quantification of oligoglia lineage cells and MBP density pre- and post-exercise intervention. This follow-up study validated the neuroimaging findings with an in-depth histological examination of CC development from infancy to adolescence and provided several novel contributions to the field of FASD research. The MRI protocol described in Milbocker et al. (2022) offered the highest signal-to-noise ratio for in vivo scanning across development; however, it is clear that interpretation of noninvasive neuroimaging findings is augmented by histological analysis of underlying white matter histology [56].

### Summary of Major Findings

To elucidate the immediate and lasting effects of AE during the BGS on white matter development, maturation of the body of the CC was examined at four salient time points: PD 10 directly following the postnatal treatment period, PD 15 at the peak of CC development in infancy, PD 30 prior to intervention exposure and the onset of circuit refinement, and PD 45 following intervention exposure in adolescence. We did not detect any acute effects of AE on the growth of the body of the CC in either sex. However, AE rats from both sexes exhibited a transient reduction in CC growth in adolescence on PD 30 that was resolved by PD 45. Additionally, we did not detect any AE-related changes to OPC proliferation from infancy to adolescence in either sex, indicating that AE during the BGS did not prevent the formation of or deplete the OPC pool, which is integral for supporting myelin plasticity in adolescence. Notably, the exercise intervention stimulated OPC proliferation in the SI control female brain but not in the AE female brain, suggesting that AE limited the total capacity for myelin plasticity in response to certain environmental stimuli in the adolescent female brain. Moreover, we discovered that AE reduced the number of myelinating OLs in the infant and juvenile female brain. Without co-labeling with a cell death marker (i.e., caspase-9), we cannot be certain that AE leads directly to apoptosis of oligodendrocytes. However, evidence from similar animal and human studies suggests that AE dually affects OPC differentiation and OL apoptosis, contributing to an overall reduction to these cells during neurodevelopment [39,40,76]. AE-related reductions to OL number in the female brain were not detected on PD 30; however, a reduction to the OL population on PD 45 was observed and was partially mediated by the adolescent exercise intervention. This may be a function of dysregulated OPC differentiation or OL autophagy following synaptic pruning and connectome refinement. AE reduced juvenile MBP production in the female brain had the opposite effect on MBP production by PD 30 and PD 45, increasing MBP production above control levels. Notably, WR intervention returned MBP production down to control levels in females. Taken together, these results suggest that AE during the BGS in females either leads to lasting impairments to the differentiation and maturation of OPCs to myelinating OLs or that reductions to the OL population resulting from alcohol-induced apoptosis in infancy may lead to a corresponding upregulation in MBP production in an attempt to rectify atypical ensheathment due to a sustained lack of OLs during development. It is evident that dysregulated myelination may be partially resolved by increased aerobic activity in adolescence. Conversely, in the male AE brain, a reduction to the OL number was observed during the juvenile and early adolescent phases (PD 15 to PD 30). However, it was resolved by PD 45 and unaffected by adolescent exercise intervention exposure. Given that the trajectory of white matter development (due to differences in pubertal onset) naturally occurs later in the male brain [77], it is possible that the timing of the exercise intervention could have been better suited to support white matter development in the male brain. This may include increasing the duration of the intervention or beginning the intervention around PD 40. Finally, densitometric analysis of MBP production in the body of CC followed a similar trajectory in the AE male brain. 

Additionally, observed reductions in oligoglia have been negatively correlated with the production of cytokines, indicating a link between acute neuroinflammation and restricted white matter growth [76]. OPC proliferation is stimulated following neuroimmune insult. Several controversial studies have sought to determine if alcohol teratogenesis restricts proliferation of oligodendrocyte precursor cells (OPCs), which could also contribute to the observed reduction in the OL population. In the studies previously described, there was no evidence of limited OPC proliferation that could have contributed to the reduction in OLs. However, in 2017, Newville and colleagues discovered that vapor inhalation of alcohol during the BGS led to a reduction in the population of OPCs in juvenile male mice [40]. Using cre-recombinase technology, the research team found that OLs differentiating during AE (final wave of developmental oligodendrogenesis) [25] were the most vulnerable to apoptosis. Convergent with these findings, another study reported a reduction in the OL number in fetal human tissue extracted from infants with a history of AE during the first and second trimesters, prior to the BGS [76]. Interestingly, the authors also noted an upregulation in OPC proliferation that was correlated with cytokine production, likely in response to OL apoptosis during early fetal development.

In addition to modifying the survival of OLs, prenatal AE alters the proliferation, differentiation, and function of neural cells and likely glial cells through epigenetic modification. Indeed, it has been demonstrated that AE during the BGS simultaneously inhibited the expression of myelin-related genes while increasing the expression of pro-inflammatory molecules in gray matter [42]. Importantly, this process for OPC differentiation during this critical period is governed by precisely coordinated HDAC activation. Shen and colleagues have elegantly shown that inhibiting class I HDAC activity during the rodent BGS disrupts the differentiation of OPCs into OLs in CC, highlighting a sensitive period for OL development [78]. Moreover, restricting OPC maturation in vitro did not prevent the differentiation of these precursors into type II astrocytes, which indicates that HDAC activity is an essential factor of OL differentiation [79]. This study sought to investigate the immediate effect of AE during the BGS on myelin-related genes in the male and female CC for comparison to histological quantification of OLs and MBP production (Mbp) and OPCs (Pdgfra) in the same region. Immediately following postnatal treatment on PD 10, AE resulted in decreased gene expression of Mbp in the male CC compared to their suckle control analogues. While this finding did not converge with our histological analysis of OLs on PD 10, these results indicate a synergistic effect of intubation stress plus AE on OL development in the male brain. Gene expression was also measured on PD 15, at the peak of CC development in infancy. It was discovered that expression of *Pdgfra* and *Mbp* were increased in the female brain compared to the male brain with no significant interaction between postnatal treatment groups. This is in agreement with work demonstrating that females have higher levels of MBP in adulthood, mediated by pubertal hormone signaling [80]. Importantly, our results suggest that sexual dimorphisms in MBP production (and, therefore, white matter development) exist in the pre-pubescent phase. While we did not discover any specific effects of AE on these early patterns of neurodevelopment, it should be noted that AE during early gestation has been shown to alter the rodent fetal neuroimmune landscape in sex-specific ways, and ultimately contributes to hyper-reactivity of the neuroimmune to future insults throughout life in all AE rats [81]. 

Furthermore, it has been demonstrated that histone deacetylation via HDAC activity is required for temporal regulation of the maturation of OPCs [78]. Since histological analysis of the number of OPCs and OLs in infant and juvenile phases indicated that OL differentiation is inhibited by AE in this model, *Hdac* expression was evaluated at similar time points. HDAC1 and HDAC3 are Class I HDACs that are responsible for initiating the differentiation of OPCs in the CC during the rat BGS [78]. AE had no effect on the gene expression of *Hdac1* and *Hdac3* on PD 10. Following the termination of the rat BGS on PD 15, it was observed that Hdac1 expression was increased in the female brain compared to the male brain, irrespective of postnatal treatment condition. While it is possible that the presence of peripheral blood might have contributed to overall gene expression measures, we decided to quickly remove brains without prior perfusion to reduce endogenous RNase activity typically brought on by hypoxia. Additionally, recent work has shown that transcardial perfusion does not alter RT-PCR results in brain tissue [82]. Collectively, these findings confirm that the trajectory of white matter development is likely faster in the female brain compared to that in the male brain from parturition. Moreover, these results suggest that the observed reduction to the OL number on PD 10 and 15 resulting from AE is likely driven by increased apoptosis of OLs instead of limited OPC maturation. 

Alcohol teratogenesis disrupts the neuroprotective actions of neurosteroids on white matter development in a sexually-dimorphic manner; thus, future studies would benefit from neuroendocrinological evaluation of the effects of AE on neurohormone cycling from infancy to adolescence. Exposing pregnant rat dams to alcohol throughout gestation inhibits cholesterol metabolism, preventing the production of endogenous progesterone production in the fetal brain while simultaneously increasing progesterone use in the postnatal infant brain [83,84]. Progesterone administration has been shown to accelerate central nervous system myelination in rats during the BGS by stimulating the differentiation of OPCs [85,86] and has promoted remyelination in the adult rat brain by a similar mechanism, emphasizing its pivotal role in stimulating myelination across the lifespan [87]. Importantly, progesterone administration during the BGS doubles the rate of OPC differentiation in the female brain compared to their male counterparts [88]. These findings indicate that innate sexual dimorphisms in white matter development occur from parturition and are partially mediated by neurosteroid signaling. Alcohol-related reductions to progesterone synthesis and signaling during the BGS may be one factor contributing to the potential OL apoptosis that begins on PD 10 in the female brain in this study.

Refinement of the neural connectome in adolescence is inextricably linked to pubertal onset (neurosteroid signaling) in mammals. Synaptic pruning of aberrant connections and the insulation of the remaining pathways optimizes communication between brain regions to support adult cognition. It is well established that the influx of progesterone production and hormone signaling resulting from pubertal onset alters white matter cytoarchitecture and the activity of myelinating glia [87,89]. For example, CC volume is increased in the male and female brain during adolescence and is correlated with increased testosterone production in the human and rat brain [90,91]. Pubertal onset naturally occurs earlier in female mammals and fluctuations in hormone signaling are regulated by the menstrual cycle (estrus cycle in rodents). However, early-life stress and drug teratogenesis have been shown to affect the timing of pubertal onset, leading to precocial or delayed development of brain regions [92,93,94,95]. It has also been demonstrated that prenatal AE leads to precocial development of white matter tracts in late childhood [96].

## 5. Conclusions

This study presented seminal findings describing the effects of a voluntary adolescent exercise intervention on the trajectory of myelination in the female brain using a rat model of FASD. Collectively, these results show that the effectiveness of behavioral interventions for youth affected by FASD may vary by sex. Pubertal onset occurs in female rats around PD 30 and in male rats around PD 45 [77,97]. Thus, it was expected that this intervention would affect the trajectory of myelination differently in the female and male rodent brain. Histological analysis of OPC number, OL number, and MBP density pre- and post-intervention supported previous findings that alterations to oligoglia population were transient in the male brain and lasting reductions to MBP density were observed [40]. In addition, these results demonstrated an immediate positive effect of exercise intervention on mitigating alterations to MBP density in AE male rats. It is possible that connectome refinement begins with pubertal onset (after the intervention period) in the male brain, thus examination of the impact of a longer intervention period on CC myelination could be considered for future studies. In contradiction, the female brain appeared to be more vulnerable to the lasting impact of alcohol teratogenesis on white matter development and was immediately more sensitive to intervention exposure. AE rats exhibited reduced OL population and elevated density of MBP. Both structural anomalies were rectified with adolescent intervention. Notably, prenatal AE has been linked to aberrant synaptic pruning, which could account for the elevated levels of MBP in AE rats in the sedentary control condition [98,99]. Thus, our results suggest that adolescent intervention did target the second peak in myelination and was beneficial in supporting the proper development of white matter in the female FASD-affected brain. These results may be due, in part, to the greater total distance run by female rats from both postnatal treatment groups compared to their male counterparts. More research is needed to evaluate the lasting impact of adolescent exercise intervention on white matter structure and function in adult females.

Second, there is ample evidence from clinical and preclinical studies suggesting that the proper development and function of the splenium subregion of the CC is particularly vulnerable to prenatal AE [34,99,100]. Specifically, Newville and colleagues (2021) demonstrated that MBP production and myelin sheath wrapping were negatively affected in an adult mouse model of FASD [43]. Nevertheless, separate analysis of MBP density in the splenium of male and female rats in our model of FASD uncovered a transient effect of AE during the BGS on this measure. Future research is needed to assess whether there is any evidence of alteration to ensheathment wrapping in the splenium. Such results would illuminate a potential neurobiological mechanism that might contribute to the observed alcohol-related deficits in visuospatial processing and sensory integration, as well as a potential biomarker for the assessment and diagnosis of FASD affected individuals. 

Few clinical studies have implemented physical activity as an intervention for older children and youth diagnosed with FASD. Exercise intervention is free, accessible, and easily implemented into existing youth programs, which highlights the importance of this research to refine and implement this strategy to improve cognitive outcomes for youth and young adults with FASD. Pritchard Orr and colleagues (2018) assessed executive function capacity following an 8-week exercise program wherein FASD-affected and control participants were asked to participate in two 1.5-h sessions of aerobic exercise per week [101]. The research team discovered that executive function capacity was improved in FASD-affected participants immediately following and 3 weeks post-exercise intervention, suggesting the efficacy of this intervention for affected youth. However, continued research is needed to develop sex- and time-specific interventions that might better facilitate white matter development in affected youth.

## Figures and Tables

**Table 1:** Significant results from *t*-tests comparing measurements of gene expression between time points.

Independent Samples *t*-Tests								
Group	Comparison	Gene Target	Time Point 1 Mean (SD)	Time Point 2 Mean (SD)	T Statistic	*p*-Value	Cohen’s d	% Change
M SC vs. M SC	PD 10 to PD 15	*Mbp* expression	1.77 (1.56)	0.89 (0.47)	1.863	0.077	0.798	−49.72
		*Pdgfra* expression	1.63 (1.66)	0.75 (0.30)	1.8	0.087	0.771	−53.99
		*Hdac1* expression	1.07 (0.44)	0.74 (0.22)	2.286	0.033	0.979	−30.84
		*Hdac3* expression	1.75 (2.05)	0.95 (0.48)	1.312	0.204	0.562	−45.71
M SI vs. M SI	PD 10 to PD 15	*Mbp* expression	1.22 (0.70)	1.15 (0.66)	0.2	0.845	0.103	−5.74
		*Pdgfra* expression	1.29 (0.77)	0.71 (0.23)	2.079	0.058	1.076	−44.96
		*Hdac1* expression	1.14 (0.58)	0.83 (0.37)	1.303	0.212	0.633	−27.19
		*Hdac3* expression	0.99 (1.15)	1.45 (0.33)	−1.086	0.295	−0.528	46.46
M AE vs. M AE	PD 10 to PD 15	*Mbp* expression	0.52 (0.45)	0.71 (0.40)	−0.987	0.336	−0.431	36.54
		*Pdgfra* expression	0.65 (0.45)	0.60 (0.39)	0.272	0.789	0.119	−7.69
		*Hdac1* expression	0.93 (0.29)	0.68 (0.37)	1.755	0.095	0.748	−26.88
		*Hdac3* expression	1.85 (1.86)	1.39 (0.42)	0.801	0.432	0.342	−24.86
F SC vs. F SC	PD 10 to PD 15	*Mbp* expression	1.27 (0.83)	1.52 (0.60)	−0.73	0.477	−0.355	19.69
		*Pdgfra* expression	1.24 (1.01)	1.85 (1.20)	−1.127	0.277	−0.548	49.19
		*Hdac1* expression	1.12 (0.52)	1.73 (0.69)	−2.096	0.052	−0.988	54.46
		*Hdac3* expression	1.06 (0.87)	1.40 (0.59)	−0.941	0.361	−0.443	32.08
F SI vs. F SI	PD 10 to PD 15	*Mbp* expression	0.71 (0.53)	1.10 (0.64)	−1.391	0.184	−0.676	54.92
		*Pdgfra* expression	1.11 (0.98)	1.06 (0.25)	0.14	0.89	0.068	−4.5
		*Hdac1* expression	1.00 (0.62)	1.13 (0.34)	−0.54	0.596	−0.255	13
		*Hdac3* expression	1.38 (1.30)	0.97 (0.18)	0.963	0.352	0.485	−29.71
F AE vs. F AE	PD 10 to PD 15	*Mbp* expression	1.09 (0.77)	1.50 (0.98)	−0.979	0.342	−0.461	37.61
		*Pdgfra* expression	1.48 (1.01)	1.36 (0.92)	0.258	0.8	0.122	−8.11
		*Hdac1* expression	0.97 (0.48)	1.50 (0.86)	−1.616	0.126	−0.762	54.64
		*Hdac3* expression	0.83 (0.40)	1.32 (0.58)	−2.067	0.055	−0.974	59.04

**Table 2:** Results from *t*-tests comparing measurements of histological parameters between time points.

Independent Samples *t*-Tests								
Group	Comparison	Histological Parameter	Time Point 1 Mean (SD)	Time Point 2 Mean (SD)	T Statistic	*p*-Value	Cohen’s d	% Change
M SI vs. M SI (SH)	PD 10 to PD 15	OPC number	4960.83 (736.40)	4712.72 (1201.26)	0.457	0.654	0.232	n/a
		OL number	9109.29 (2634.41)	12,394.73 (4152.54)	−1.858	0.082	−0.898	n/a
		MBP density body of corpus callosum	n/a	n/a	n/a	n/a	n/a	n/a
		MBP density splenium of corpus callosum	n/a	n/a	n/a	n/a	n/a	n/a
	PD 15 to PD 30	OPC number	4712.72 (1201.26)	30,261.80 (13,127.84)	−6.442	<0.001	−2.815	542.13
		OL number	12,394.73 (4152.54)	55,843.40 (11,610.49)	−11.201	<0.001	−5.088	350.54
		MBP density body of corpus callosum	0.43 (0.09)	0.44 (0.08)	−0.261	0.797	−0.114	n/a
		MBP density splenium of corpus callosum	0.22 (0.05)	0.29 (0.05)	−3.4	0.004	−1.62	n/a
	PD 30 to PD 45	OPC number	30,261.80 (13,127.84)	49,201.27 (15,212.47)	4.13	0.002	1.25	62.58
		OL number	55,843.40 (11,610.49)	50,416.18	−1.1	0.315	−0.319	n/a
		MBP density body of corpus callosum	0.44 (0.08)	0.42 (0.11)	−0.667	0.52	−0.193	n/a
		MBP density splenium of corpus callosum	0.29 (0.05)	0.28 (0.07)	−0.37	0.73	−0.12	n/a
M SI vs. M SI (WR)	PD 30 to PD 45	OPC number	30,261.80 (13,127.84)	51,705.60 (18,540.18)	3.66	0.005	1.16	70.86
		OL number	55,843.40 (11,610.49)	64,613.67 (31,081.10)	0.85	0.42	0.28	n/a
		MBP density body of corpus callosum	0.44 (0.08)	0.42 (0.04)	−1.4	0.2	−0.5	n/a
		MBP density splenium of corpus callosum	0.29 (0.05)	0.35 (0.09)	2.1	0.07	0.7	n/a
M AE vs. M AE (SH)	PD 10 to PD 15	OPC number	4453.00 (771.65)	4727.88 (242.38)	−1.109	0.286	−0.449	31.82
		OL number	9627.91 (1124.10)	10,500.11 (2173.88)	−1.159	0.262	−0.521	n/a
		MBP density body of corpus callosum	n/a	n/a	n/a	n/a	n/a	n/a
		MBP density splenium of corpus callosum	n/a	n/a	n/a	n/a	n/a	n/a
	PD 15 to PD 30	OPC number	4727.88 (242.38)	19,304.27 (8424.22)	−4.85	<0.001	−2.26	308.31
		OL number	10,500.11 (2173.88)	43,716.5 (10,411.86)	−9.36	<0.001	−4.13	316.34
		MBP density body of corpus callosum	0.33 (0.06)	0.40 (0.05)	−2.6	0.02	−1.21	21.21
		MBP density splenium of corpus callosum	0.15 (0.06)	0.23 (0.05)	−3.4	0.004	−1.6	53.33
	PD 30 to PD 45	OPC number	19,304.27 (8424.22)	48,456.56 (13,015.88)	6.72	<0.001	2.24	151.01
		OL number	43,716.5 (10,411.86)	61,180.56 (22,375.24)	2.34	0.047	0.78	39.95
		MBP density body of corpus callosum	0.40 (0.05)	0.37 (0.11)	−0.877	0.4	−0.28	n/a
		MBP density splenium of corpus callosum	0.23 (0.05)	0.29 (0.14)	1.35	0.21	0.43	n/a
M AE vs. M AE (WR)	PD 30 to PD 45	OPC number	19,304.27 (8424.22)	48,697 (12,252.38)	6.35	<0.001	2.4	152.26
		OL number	43,716.5 (10,411.86)	49,567 (16,860.13)	0.98	0.34	0.35	n/a
		MBP density body of corpus callosum	0.40 (0.05)	0.52 (0.15)	2.3	0.06	0.87	n/a
		MBP density splenium of corpus callosum	0.23 (0.05)	0.28 (0.08)	1.36	0.24	0.61	n/a
F SI vs. F SI (SH)	PD 10 to PD 15	OPC number	4481.30 (1147.03)	4844.90 (1244.13)	−0.679	0.505	−0.304	n/a
		OL number	9653.100 (2390.77)	12,409.10 (1943.46)	−2.829	0.011	−1.265	28.55
		MBP density body of corpus callosum	n/a	n/a	n/a	n/a	n/a	n/a
		MBP density splenium of corpus callosum	n/a	n/a	n/a	n/a	n/a	n/a
	PD 15 to PD 30	OPC number	4844.90 (1244.13)	25,258.90 (11,801.67)	−5.44	<0.001	−2.43	421.35
		OL number	12,409.10 (1943.46)	44,692 (8861.01)	−11.25	<0.001	−5.03	260.16
		MBP density body of corpus callosum	0.36 (0.06)	0.47 (0.07)	−3.77	0.002	−1.73	30.55
		MBP density splenium of corpus callosum	0.18 (0.10)	0.30 (0.06)	−3.1	0.007	−1.4	66.67
	PD 30 to PD 45	OPC number	25,258.90 (11,801.67)	30,564.29 (9962.98)	1.41	0.21	0.53	n/a
		OL number	44,692 (8861.01)	46,855.88 (21,523.32)	0.28	0.78	0.1	n/a
		MBP density body of corpus callosum	0.47 (0.07)	0.38 (0.05)	−4.76	0.005	−1.95	n/a
		MBP density splenium of corpus callosum	0.30 (0.06)	0.27 (0.08)	−1	0.37	−0.41	−19.15
F SI vs. F SI (WR)	PD 30 to PD 45	OPC number	25,258.90 (11,801.67)	51,238.60 (17,597.21)	4.68	0.001	1.48	102.85
		OL number	44,692 (8861.01)	57,836.44 (8534.98)	4.62	0.002	1.54	29.41
		MBP density body of corpus callosum	0.47 (0.07)	0.41 (0.05)	−3.76	0.004	−119	−12.77
		MBP density splenium of corpus callosum	0.30 (0.06)	0.35 (0.13)	1.2	0.26	0.38	
F AE vs. F AE (SH)	PD 10 to PD 15	OPC number	3769.18 (954.78)	5615.14 (462.72)	−4.68	<0.001	−2.358	48.98
		OL number	7282.78 (1448.05)	10,884.40 (3066.90)	−3.209	0.005	−1.474	49.45
		MBP density body of corpus callosum	n/a	n/a	n/a	n/a	n/a	n/a
		MBP density splenium of corpus callosum	n/a	n/a	n/a	n/a	n/a	n/a
	PD 15 to PD 30	OPC number	5615.14 (462.72)	25,017 (7707.62)	−7.1	<0.001	−3.42	345.52
		OL number	10,884.40 (3066.90)	39,506.75 (14,860.75)	−5.36	<0.001	−2.83	263
		MBP density body of corpus callosum	0.27 (0.15)	0.54 (0.11)	−4.2	<0.001	−2	100
		MBP density splenium of corpus callosum	0.11 (0.04)	0.34 (0.08)	−7.22	<0.001	−3.61	209.1
	PD 30 to PD 45	OPC number	25017 (7707.62)	34,108.56 (8416.51)	3.24	0.01	1.08	36.34
		OL number	39,506.75 (14,860.75)	38,872.88 (7914.29)	−0.23	0.83	−0.08	n/a
		MBP density body of corpus callosum	0.54 (0.11)	0.60 (0.12)	1.38	0.21	0.49	n/a
		MBP density splenium of corpus callosum	0.34 (0.08)	0.35 (0.04)	0.43	0.69	0.16	n/a
F AE vs. F AE (WR)	PD 30 to PD 45	OPC number	25,017 (7707.62)	35,028.90 (9536.40)	3.32	0.009	1.05	40.02
		OL number	39,506.75 (14,860.75)	46,393.70 (10,521.17)	2.07	0.07	0.66	n/a
		MBP density body of corpus callosum	0.54 (0.11)	0.41 (0.14)	−2.95	0.02	−0.93	−24.07
		MBP density splenium of corpus callosum	0.34 (0.08)	0.32 (0.14)	−0.57	0.58	−0.18	n/a

## Data Availability

The raw data supporting the conclusions of this article will be made available by the authors, without undue reservation.
